# Herpes Infections and Dementia: Rebutting Alternative Fact

**DOI:** 10.1007/s13311-018-00700-5

**Published:** 2019-01-07

**Authors:** Richard Lathe, Nian-Sheng Tzeng, Ruth Itzhaki

**Affiliations:** 1 Division of Infection and Pathway Medicine, University of Edinburgh, Little France, Edinburgh, UK; 2 Department of Psychiatry, Tri-Service General Hospital, School of Medicine, National Defense Medical Center, Taipei, Taiwan, Republic of China; 3 Nuffield Department of Clinical Neurosciences, John Radcliffe Hospital, Oxford, UK; 4 School of Biological Sciences, University of Manchester, Oxford Road, Manchester, UK

**Keywords:** Alzheimer's disease, Herpes simplex virus, Acyclovir, APOE

## Abstract

Recent commentary in *Neurotherapeutics* by Nath critically addresses the earlier report by Tzeng et al. that aggressive antiviral treatment (AVT) against herpes simplex virus (HSV) was associated with a later decrease in the incidence of Alzheimer’s disease (AD). Nath raises issues that we respond to: we point out that (i) the treated group (probably with severe infection) is likely to harbor genetic risk alleles that predispose to both AD and HSV infection—the potential treatment bias cited by Nath would support (rather than challenge) the preventive effect of AVT; (ii) HSV is well known to establish persistent infection in the brain; and (iii) current AVT compounds used to combat herpes viruses are highly specific for this class of viruses. Instead of “alternative fact,” the findings of Tzeng et al. argue in favor of clinical trials of AVT in AD.

**Electronic supplementary material:**

The online version of this article (10.1007/s13311-018-00700-5) contains supplementary material, which is available to authorized users.

The recent concise commentary by Avindra Nath [1] discusses the report by one of us (Tzeng et al. [2]), in a recent issue of *Neurotherapeutics* that addressed a potential protective role for antiviral therapy (AVT) in Alzheimer’s disease (AD). In this retrospective study employing the Taiwan National Health Insurance Database, Tzeng et al. reported that short-term AVT of patients with overt herpes virus infections was followed by a long-term reduction in the subsequent incidence of AD over the following decade ([2]; discussed in [3]). In his commentary, Dr Nath queries the results of this study (“alternative fact”), and concludes that it might merely be safe to treat “patients who develop herpes virus infections with appropriate antiviral drugs” [1], a proposal wisely consistent with current practice. Although the commentary makes interesting points, other issues appear to be misconstrued and warrant urgent rectification, which we offer in the hope of clarifying the situation.

Dr Nath is correct is in pointing out that the Tzeng et al. study is retrospective in nature, and thus raises issues concerning proper diagnosis in the absence of neuroimaging or postmortem pathological analysis. However, the Taiwan database employs an accredited diagnostic instrument (International Classification of Disease, Ninth Revision, Clinical Modification; ICD-9-CM), and thus is unlikely to be a major confounding factor.

Dr Nath further points out that there was also a lack of identification of genetic risk factors. We agree that this latter is extremely important because diverse immune-related genes are associated both with infection and AD development. Centrally, *ε4* alleles of the *APOE* gene are susceptibility factors not only for AD but also for several infectious diseases. The *ε4* allele is known to increase susceptibility to infection damage by, among others, herpes simplex virus type 1 (HSV1) [4, 5], HSV2 [6, 7], *Chlamydia pneumoniae* [8], *Chlamydia*-associated arthritis [9], and HIV [10]. Some other AD risk alleles are also known to predispose to infectious disease; for example, genetic variants in *PILRA*, a receptor for HSV1 glycoprotein B (that governs HSV1 viral entry into cells), are associated with AD [11]. Therefore, the population studied in the Tzeng et al. report (acute HSV1/2 infection) was likely to have been enriched in AD risk alleles, including *APOE ε4*, and thus may have been inherently at increased risk of later AD development. No genomic studies were performed on patients in the Tzeng et al. study, and although we do not believe that this detracts from the potential protective role of AVT reported by Tzeng et al., we agree (as discussed further below) that such genetic predisposition could potentially underlie positive associations (possibly circumstantial) between AD and different infectious conditions including herpes zoster ophthalmicus [12], varicella zoster [13], periodontal disease [14–16], *Borrelia burgdorferi* [17], and *Chlamydophila pneumoniae* [18].

We highlight this genetic factor because it must be carefully borne in mind when considering the next caveat listed by Nath [1]—“selection bias for treatment.” The Tzeng et al. [2] study compared patients with acute HSV-induced lesions (predominantly orolabial and genital) who received AVT *versus* those who were not treated. It could be argued that the patients who received AVT were likely to have suffered from more severe lesions than those who went untreated. In consequence, the AVT group (likely with more serious lesions) may have been biased towards inclusion of individuals with a genetic predisposition to AD.

Our central argument, therefore, is that the AVT group might have been at increased (rather than decreased) risk of later AD development. By contrast, it was precisely the AVT group that showed a dramatic reduction in later AD development. The potential treatment bias cited by Nath argues in exactly the reverse direction. Thus, it is even more remarkable that their treatment with AVT apparently prevented most (ca 90%) cases of AD development in the decade that followed. Treatment bias, if it exists, would therefore entirely support (rather than challenge) the reality of the preventive effect of AVT.

An additional major issue in Nath’s commentary also calls for clarification. He states that “one would have to postulate that the virus establishes a chronic or persistent infection in the brain to cause cognitive deficits. However, given what we know about these viruses, this would be highly unlikely” [1]. We must point out that this is not correct.

Seropositivity for HSV1 and HSV2 increases over our lifetime: HSV infection is generally acquired during postnatal life, and HSV seropositivity increases with age in the USA [19] and Europe [20], as well as in Taiwan [21], and by age 50 years, the majority of the population harbors HSV1 and/or HSV2 (often in addition to other herpes viruses such as cytomegalovirus and Epstein–Barr virus). Following primary infection (generally of epithelial cells) that can be symptomatic or asymptomatic, HSV1 enters a silent (latent) state (reviewed in [22, 23]) principally in neurons in peripheral ganglia and other tissues, from where it can intermittently reactivate. In the nervous system, HSV1 can latently persist for decades, notably in the trigeminal ganglia [24–27], but can invade many brain regions. HSV1 receptors are most abundantly expressed in the hippocampus [28], a site of early degeneration in AD. Indeed, HSV1 DNA can be detected in several brain regions including the hippocampus, both of control individuals and AD patients [29, 30], but levels are upregulated in AD brain, as recently confirmed for HSV1/2 as well as for human herpes viruses (HHV) 6 and 7 [31]. The pathological signature of AD, Abeta protein, is induced by HSV1 infection [32] and is increasingly recognized as an antimicrobial factor ([33], reviewed in [34]) that exerts potent antiHSV1 effects [35, 36]. Although *de novo* brain invasion with HHV following disease onset cannot be ruled out [37], this is not the case for HSV1 that is widely detected in both AD and control brain tissues [30, 31, 38]. There are also suggestions that a declining immune system in the elderly might predispose to virus reactivation: the immune system undergoes systemic changes over a lifetime (e.g., [39]) and reactivation of HSV1 has been reported to take place as a function of age [40, 41]. Diverse types of evidence for reactivation of HSV1 in human brain have been described in detail [42]. The available data thus manifestly rebut Nath’s view that “a chronic or persistent infection in the brain ... would be highly unlikely.”

This indirectly answers the question raised by Nath—“why would herpes virus infection in the genitalia or lips lead to dementia”—in short, it may not (or not directly). Instead, reactivation of epithelial HSV1 might be accompanied (particularly in genetically predisposed individuals) by reactivation in other tissues including the brain, that then initiates the lesions that, over the following decades, culminate in clinical AD. Alternatively (or in addition), in cases of *de novo* orogenital infections, increased peripheral virus load and/or inflammation might increase the likelihood that virus enters the brain [3].

A final issue raised by Nath concerns the apparent nonspecificity of the association between infection, AVT, and AD. He very properly highlights that several types of infectious agent have been associated with AD, and that minor infections of the elderly can be associated with cognitive decline. However, this nonspecificity does not extend to AVT. Current AVT compounds used to combat herpes viruses are highly specific for this class of viruses: these agents (e.g., acyclovir, ganciclovir, penciclovir) are nucleoside analogs that require phosphorylation by the viral thymidine kinase enzyme (and recognition of the ensuing triphosphates by viral DNA polymerases) for inhibition of virus proliferation. These compounds are poorly recognized by the corresponding enzymes of other virus classes and display much less or no antiviral activity against them (e.g., vaccinia virus or even veterinary herpes viruses) [43, 44]. There is evidence that ganciclovir does have inhibitory activity against adenovirus [45], but this is not the first-line antiherpetic drug, and others in the class are ineffective against adenoviruses. There have been suggestions that acyclovir might have some activity against HIV by targeting the reverse transcriptase enzyme [46], but virus-inhibitory concentrations are 10–100-fold higher than those for herpes viruses, and acyclovir failed to prevent HIV transmission [47]. Therefore, although inhibition of a so far unidentified virus class cannot yet be ruled out, it is most likely that the protective efficacy of AVT observed by Tzeng et al. is through selective targeting of one or more herpes viruses (although the precise virus type remains to be established).

To conclude, Dr Nath has clearly not overlooked the potential implications of the Tzeng et al. report. However, we argue that, far from representing “alternative fact,” the findings of the Tzeng et al. argue strongly in favor of clinical trials of AVT in AD.

## Required Author Forms

Disclosure forms provided by the authors are available with the online version of this article.

## Electronic Supplementary Material

ESM 1 (PDF 1261 kb)

ESM 2 (PDF 1267 kb)

ESM 3 (PDF 1266 kb)
